# Computed Tomography of Contemporary Occupational Lung Disease: A Pictorial Review

**DOI:** 10.3390/diagnostics14161786

**Published:** 2024-08-16

**Authors:** Jimin Lee, Marie Bambrick, Ambrose Lau, Susan M. Tarlo, Micheal McInnis

**Affiliations:** 1 Temerty Faculty of Medicine, University of Toronto, Toronto, ON M5S 1A8, Canada; 2 Department of Medical Imaging, Faculty of Medicine, University of Toronto, Toronto, ON M5S 1A1, Canada; 3 Department of Medical Imaging, Toronto General Hospital, Toronto, ON M5G 2C4, Canada; 4 Division of Respirology, University Health Network, Toronto, ON M5T 2S8, Canada

**Keywords:** occupational lung disease, pneumoconiosis, asbestosis, silicosis, hypersensitivity pneumonitis, work-related asthma

## Abstract

Occupational lung disease remains one of the most common work-related illnesses and accounts for most deaths from occupational illness. Occupational lung diseases often have delayed manifestation over decades and nonspecific clinical presentations, making it challenging for clinicians to promptly identify the disease and implement preventive measures. Radiologists play a crucial role in identifying and diagnosing occupational lung diseases, allowing for removal of the exposure and early medical intervention. In this review, we share our clinical and radiologic approach to diagnosing occupational lung disease and its subtypes. A collection of sample cases of occupational lung diseases commonly encountered in the modern era at a large Canadian university hospital is included to facilitate understanding. This review will provide radiologists with valuable insights into recognizing and diagnosing occupational lung diseases.

## 1. Introduction

Occupational lung disease encompasses a broad range of pulmonary disorders that arise due to the inhalation of organic and inorganic particulates or gases in the workplace. Despite improvements in workplace safety and personal protective equipment (PPE), occupational lung disease remains one of the most common work-related illnesses and accounts for most deaths from occupational illness [1].

Awareness of occupational lung disease remains important in Canada despite a decreasing incidence commensurate with strengthening health and safety guidelines [2]. These diseases often clinically manifest decades following exposure, and prohibited substances may still have lingering effects to this day [3]. Additionally, occupational lung disease continues to evolve as new conditions arise in the modern era with new workplace exposures. Canada has a large population of first-generation immigrants who may have been exposed in workplace environments that are less strictly regulated [4].

Central to the challenge in diagnosing occupational lung disease is the nonspecific symptomatology, and yet these diseases often have stereotypical imaging features [5]. Radiologists thus play a key role in recognizing occupational lung diseases and making an early diagnosis. Early intervention is key to managing occupational lung diseases, which principally involves timely removal of the exposure [6].

In this review, we will share our approach to the diagnosis of occupational lung disease with a focus on the imaging features that best assist the radiologist in making a diagnosis. We further present a collection of images related to occupational lung diseases, both old and new, encountered in the modern era at a large Canadian university hospital.

## 2. Clinical Presentation

Occupational lung disease includes multiple diseases that commonly mimic non-occupational lung diseases. These diseases include pneumoconioses, hypersensitivity pneumonitis, and work-related asthma, as well as less commonly recognized entities such as occupational COPD, bronchiolitis, acute pneumonitis, and lung cancer. Pneumoconioses and occupational hypersensitivity pneumonitis are largely differentiated from non-occupational lung diseases based on the exposure history [2]. Pneumoconioses are lung diseases caused by the inhalation of different dusts that are most commonly occupational in origin [7]. Hypersensitivity pneumonitis is caused by exposure to organic or low-molecular weight inorganic dusts which may be occupational or environmental in origin [8].

The clinical evaluation of occupational lung disease relies on a careful assessment of patient symptoms, particularly their onset and relationship to an occupational exposure [9]. Clinicians must maintain a high level of clinical suspicion in patients presenting with respiratory symptoms and screen for occupational exposure history, physical examination, pulmonary function testing, and medical imaging [9].

The core principle in the management of occupational lung disease is prevention of disease by avoiding exposure to substances that can cause disease (primary prevention). When this is not possible, exposure should be minimized by occupational hygiene measures that may include respiratory protective devices. Medical surveillance may allow early detection of disease. Once disease has been identified, removal of the patient from the identified exposure is a key component of management in addition to other management of the disease [6]. Radiologists play a crucial role in diagnosis and management by delineating occupational lung diseases and potential exposures based on imaging presentation.

## 3. Work-Related Asthma

Work-related asthma is principally a clinical disease without specific radiologic findings but is important to discuss, as it is a common occupational lung disease accounting for around 16% of new adult asthma diagnoses [1,10]. There has been a growing realization of the prevalence of work-related asthma across various industries, including teaching and service sectors, associated with mould, construction dust, printing agent, and cleaning solution exposure [11,12,13]. Recently, there was a case of a cannabis cultivation facility worker that suffered from occupational asthma and experienced serious exacerbations that resulted in death [14].

Work-related asthma is a broad term that encompasses subtypes: work-exacerbated asthma, irritant-induced asthma, and immunologic occupational asthma [15]. These disease distinctions are important to make due to the difference in symptom onset following substance exposure; irritant-induced asthma typically manifests within 24 h of exposure, while immunologic occupational asthma is often delayed by anywhere from weeks to years in onset [15]. Work-related asthma is radiologically indistinguishable from asthma with other causes, and clinical assessment with a thorough occupational history is crucial to its diagnosis [16].

## 4. Pneumoconiosis

### 4.1. Silicosis

Silicosis is the leading cause of occupational pneumoconiosis, constituting 39% of all pneumoconiosis cases [17]. Patients are often asymptomatic, and the diagnosis is often detected with surveillance or incidental chest imaging. However, if it is associated with fibrosis or if it is complicated by infection, silicosis may present with cough, dyspnea, and/or systemic symptoms, such as fever and weight loss [18]. Silicosis is caused by the inhalation of crystalline silicone dioxide (silica), found in stone and sand [17]. Stone cutting, mining, sandblasting, and quarrying are common occupations where patients may be exposed to silica dust [19]. Although the overall incidence of silicosis has decreased in many countries, there has been a re-emergence of cases in specific workplace environments such as the fashion industry due to manufacturing of sandblasted denim and the production and use of engineered artificial stone, such as in use for countertops [20,21]. Silica exposure can lead to a variety of conditions, including simple silicosis, complicated silicosis (progressive massive fibrosis), acute silicosis (silicoproteinosis), and accelerated silicosis [22].

Simple silicosis typically presents 10–20 years after the occupational exposure. In most cases, patients are asymptomatic and pulmonary function tests may be normal [18]. CT findings of silicosis include profuse small nodules measuring 2–5 mm with a posterior and upper lung zone predominance. The small nodules can coalesce in the subpleural lung to form pseudo-plaques along the pleural surface [23]. Calcification can occur both in the lung nodules and lymph nodes, which is classically described as having an eggshell appearance [24]. Although classic, most calcified lymph nodes in silicosis will not have the eggshell-like appearance.

**Figure 1:**
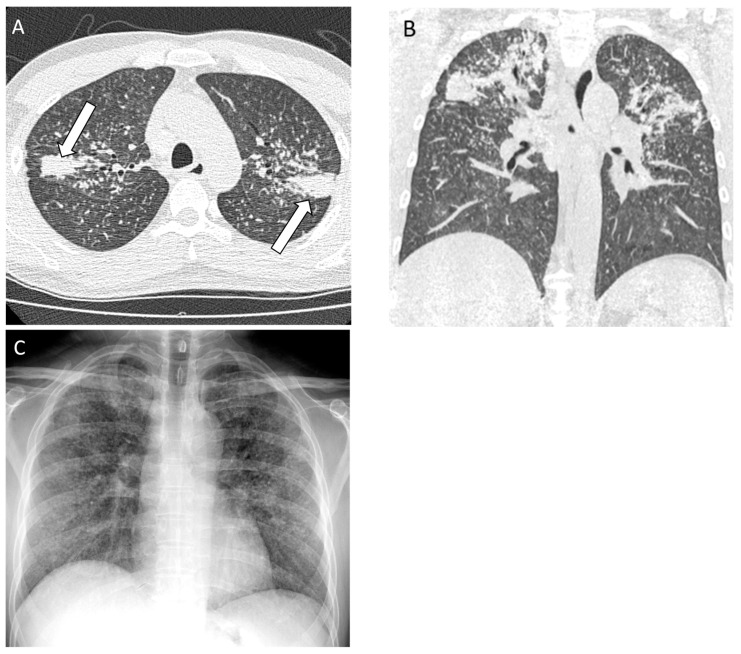
A 45-year-old non-smoking male, previously a stone mason and countertop stone cutter, diagnosed with complicated silicosis and progressive massive fibrosis. (**A**) Axial computed tomography image shows conglomerate bilateral upper zone masses with associated architectural distortion consistent with progressive massive fibrosis (white arrows); (**B**) coronal image demonstrates the upper lung zone predominance; (**C**) chest radiograph demonstrates profuse small circumscribed pulmonary nodules with an upper lung zone predominance.

Complicated silicosis occurs when progressive massive fibrosis takes place [22]. In contrast to simple silicosis, patients with complicated silicosis will often present clinically with a decline in lung function and restrictive physiology [23]. The hallmark of progressive massive fibrosis is the coalescence of silicotic nodules towards the hilum, forming conglomerate mass-like lesions, with the peripheral lung showing features of fibrosis with traction and paracicatricial emphysema [25]. Similar to simple silicosis, calcification is observed on computed tomography (CT), and the peripheral lung may continue to show a background of small nodules (Figure 1).

Accelerated silicosis is a more rapidly progressive presentation of silicosis, occurring within 10 years of exposure [22]. The imaging findings are similar to those seen in complicated silicosis [26]. Importantly, accelerated silicosis is becoming increasingly common in recent years due to the popularity of artificial stones, such as quartz countertops, for their strong non-porous properties compared to natural stones [27]. Artificial stones have high silica content, reaching up to 90%. The method of cutting artificial stone is also a crucial factor in the generation of silica dust; dry cutting methods generate more silica dust than wet cutting methods [28].

Silicoproteinosis represents a rare, acute presentation related to silica exposure where patients are typically exposed to very high concentrations of silica dust [3]. Such instances may occur in workers in an enclosed space without PPE usage, most classically with sandblasting, and have been reported more recently in a high proportion of young workers sandblasting denim [21]. CT findings of silicoproteinosis are predominantly widespread ground glass opacities and consolidation correlating with alveolar filling with proteinaceous material [26]. Interstitial lines may be present, including the crazy-paving appearance of intralobular septal thickening [29].

Silicosis has potential complications that radiologists must be aware of. Silicosis is an independent risk factor for the development of emphysema, lung cancer, and tuberculosis infection [23]. The presence of progressive massive fibrosis found in complicated and accelerated silicosis can mimic lung cancer. An additional T2 weighted image on magnetic resonance imaging where progressive massive fibrosis appears as low-signal intensity and lung cancer as intermediate- or high-signal intensity may support in distinguishing between the two conditions [30].

### 4.2. Coal Worker’s Pneumoconiosis

Coal worker’s pneumoconiosis (CWP), also known as black lung disease, results from the inhalation of inorganic coal dust by workers in coal mines [31]. Since the recognition of the respiratory harm and subsequent development of coal dust concentration regulations in coal mines to minimize workers’ exposure in 1990, the incidence of CWP has decreased in North America [31,32]. However, there have been increased cases of CWP in the United States in the past few decades, despite the strict dust concentration regulations; nano-sized coal dust particles are hypothesized to be the culprit [33].

CWP is characterized by a more varied exposure compared to silicosis due to the diverse mineral structure and mining techniques employed in coal mining. Typically, patients with CWP have also been exposed to silica, and the radiologic presentation is almost identical with a correlate of simple or complicated CWP, although lymph node calcification is less common in CWP than silicosis [34]. The treatment is similar, and histological examination aids in delineating the two diseases [7].

### 4.3. Asbestos-Related Lung Disease

Asbestos-related lung disease results from the inhalation of asbestos fibres. These fibres are classified in two groups, serpentines and amphiboles, wherein amphibole fibres are associated with higher morbidity [35,36]. Asbestos may be encountered in many industries, including construction for its insulation capabilities, shipyard trades, and in the automobile industry for friction material in brake pads [37]. Although the use of asbestos is decreasing in many countries, it is still routinely used in the developing world [37]. In Canada, asbestos use, including importing and sales, was prohibited in 2018, and the last asbestos mines closed in 2011 [38,39]. The United States prohibited chrysolite asbestos, the only type of asbestos currently used for chlor-alkali production, in March 2024 after the recognition of exposure hazards [40]. Health consequences of asbestos exposure commonly manifest 15 years or more after exposure; thus, asbestos-related disease may be encountered in modern practice [36,41].

**Figure 2:**
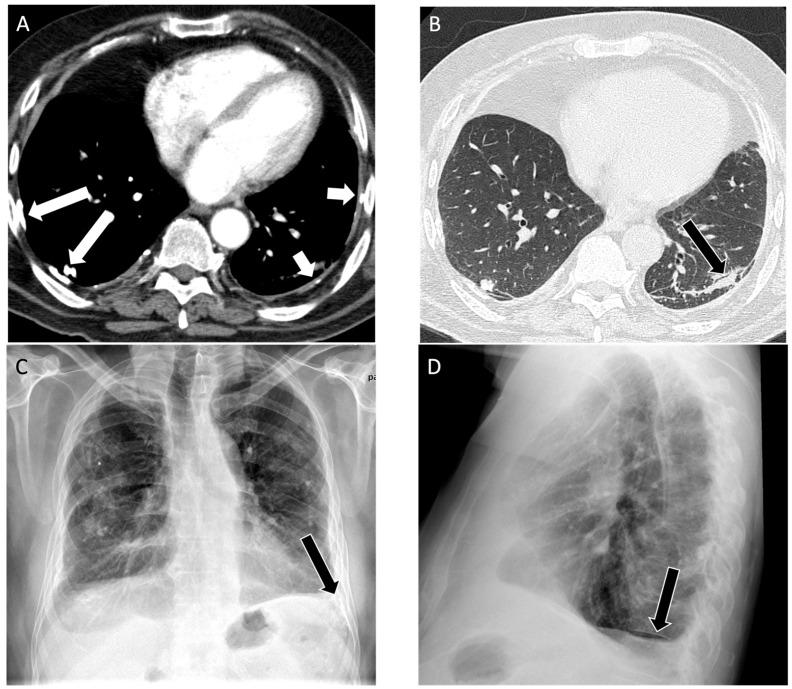
A 73-year-old male with a history of remote 16 pack year smoking history and asbestos exposure as a retired plumber working on pipe and broiler insulation, diagnosed with asbestos-related pleural disease. (**A**) Axial computed tomography shows asbestos plaques on the right (long arrows) and diffuse pleural thickening on the left (short arrows) on the soft tissue window; (**B**) on the lung window, a subpleural band opacity is seen in the left lower lobe (black arrow); (**C**) posteroanterior view and (**D**) lateral view chest radiographs show diffuse pleural thickening with calcification, best appreciated by costophrenic angle blunting (black arrows).

Asbestos-related pleural effusions may be the earliest manifestation of asbestos exposure, occurring in the first decade [42]. Effusions can be unilateral or bilateral and are generally small in volume; thoracentesis typically reveals exudative fluid [42]. Diffuse pleural thickening is not common and may be an isolated finding or may be the sequela of an asbestos-related pleural effusion. In contrast to the pleural plaques that appear later in the course of the disease, diffuse pleural thickening typically manifests as broad areas of smooth and mild thickening which extend over a length greater than 8 cm without calcification (Figure 2) [43]. Diffuse pleural thickening may cause lung volume loss and thus may be symptomatic with a restrictive pattern on spirometry [41]. Indeed, rounded atelectasis is seen in this population, and while presentation may be variable, a confident diagnosis of rounded atelectasis can be made when there is a rounded opacity abutting pleural thickening with a classic comet-tail sign of the bronchovascular bundle coursing into the area of rounded atelectasis [44].

Pleural plaques represent the classic and most common manifestation of asbestos exposure [45]. These plaques, typically characterized as focal areas of calcified or non-calcified pleural thickening measuring several centimeters in diameter and millimeters in thickness, are later signs of asbestos-related lung disease (Figure 2) [45]. Plaques are most commonly bilateral and can be seen along all pleural surfaces, though the diaphragm is a classic presentation with sparing of the apices and costophrenic angles [46]. Despite the commonality of pleural plaques in asbestos disease manifestation, these plaques are benign and were found to not increase the risk of lung cancer [47]. Radiologists should maintain a high index of suspicion for plaques with atypical features, such as nodular morphology with or without calcification and invasion to surrounding lymph nodes or the contralateral lung, which may suggest mesothelioma.

**Figure 3:**
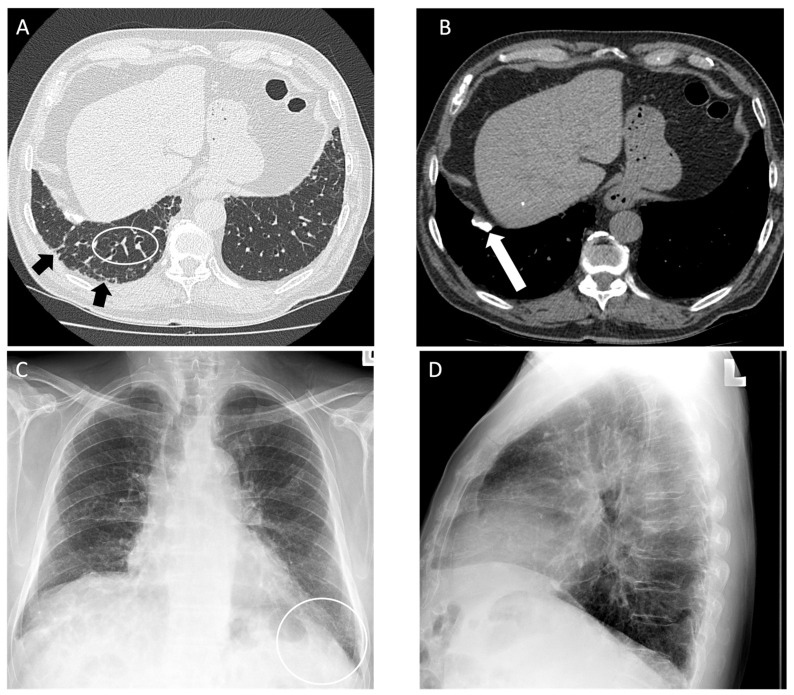
An 83-year-old male with 75 pack year smoking history and asbestos exposure from construction work, diagnosed with asbestosis. (**A**) Axial computed tomography image demonstrates subpleural reticulation (black arrows) and mild traction bronchiectasis (white circle) of right lung in keeping with mild asbestosis; **(B**) mediastinal windows demonstrate a calcified pleural plaque along the right diaphragm (white arrow); (**C**) posteroanterior and (**D**) lateral chest radiograph demonstrate mild reticular markings in the basal lung consistent with mild fibrosis, best appreciated in the left lateral costophrenic angle (white circle).

Asbestosis is defined as interstitial pulmonary fibrosis from asbestos exposure. There is often a history of significant and prolonged occupational exposure, and there is an established relationship between the exposure history and the amount of pulmonary fibrosis [48]. Like fibrosis of a usual interstitial pneumonia pattern, asbestosis manifests as a basal and subpleural predominance of reticulation, traction bronchiectasis, and honeycombing on CT [48]. Ground glass opacity may be present in the areas of reticulation, likely representing microscopic fibrosis [49]. In addition to the findings of usual interstitial pneumonia, patients with asbestosis may have subpleural curvilinear opacities and parenchymal band opacities [50]. The presence of other stigmata of asbestos exposure, such as pleural plaques, indicates a likely diagnosis of asbestosis rather than other interstitial lung diseases (Figure 3).

**Figure 4:**
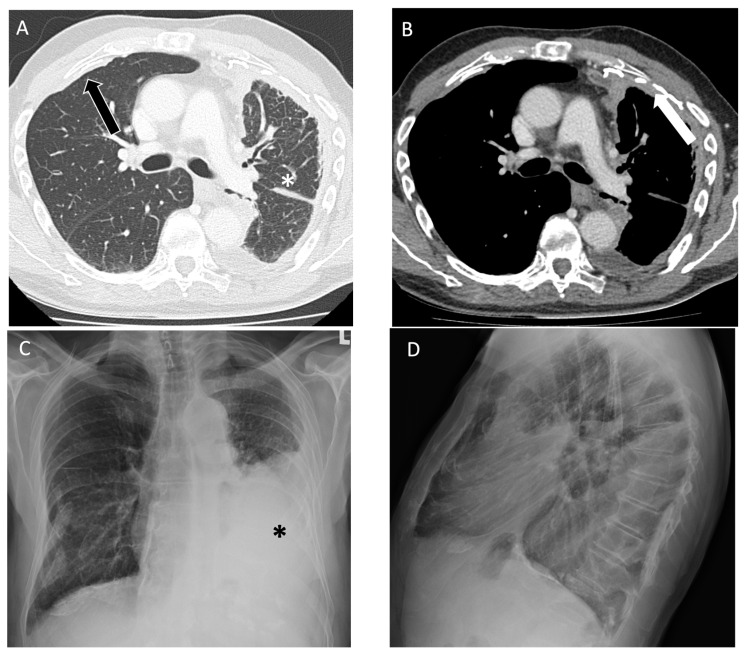
An 89-year-old non-smoking male with asbestos exposure from construction work, diagnosed with epithelioid mesothelioma, later confirmed on video-assisted thoracoscopic surgery biopsy. (**A**) On axial computed tomography, there is a partly calcified pleural plaque anteriorly on the right (black arrow) and volume loss in the left lung (asterisks) related to a rind of pleural thickening; (**B**) on the left, the rind of pleural thickening (arrow) has a nodular appearance consistent with mesothelioma and there is a small pleural effusion after thoracentesis that drained 1.2 L of fluid; follow-up (**C**) posteroanterior and (**D**) lateral view radiographs one week later demonstrate a reaccumulated moderate-to-large size pleural effusion opacifying the left mid-to-lower lung zone (asterisks).

Mesothelioma is an aggressive neoplasm of the mesothelium, which typically presents at a late oncologic stage, around 20 to 40 years after asbestos exposure, and often the exposure is relatively low [51]. Mesothelioma most commonly arises from the pleura and manifests as various types of pleural thickening (nodular, lobular) rather than benign smooth pleural thickening (Figure 4) [51]. Pleural effusion and plaques are common; mesothelioma must be ruled out for patients presenting with dyspnea and chest wall pain, as these symptoms are manifestations of pleural effusion and chest wall invasion [52,53].

### 4.4. Hard Metal Lung Disease

**Figure 5:**
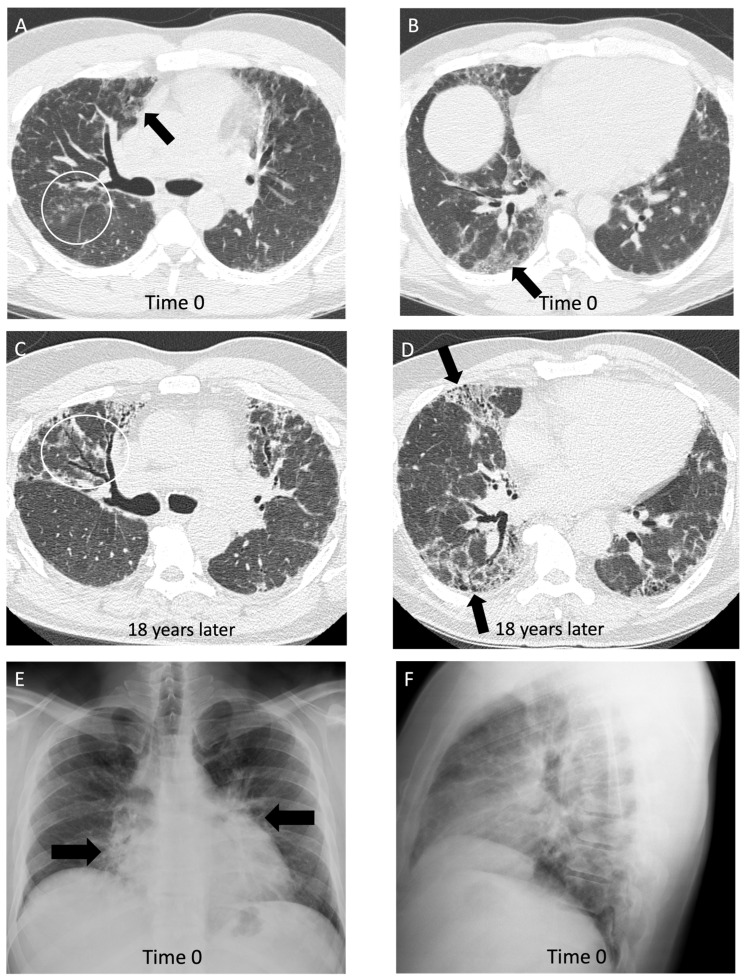

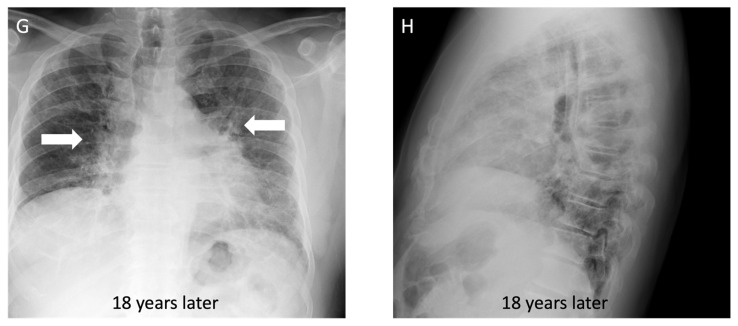
A 43-year-old non-smoking male with 15-year history of tungsten carbide exposure as a metal grinding tool maker, diagnosed with hard metal lung disease, which was confirmed with surgical lung biopsy. (**A**,**B**) Axial computed tomography images demonstrate patchy ground glass opacities (arrows) and small centrilobular nodules (white circle) with minimal architectural distortion and traction bronchiectasis; (**C**,**D**) computed tomography images 18 years later demonstrate long-term evolution of fibrosis, with development of traction bronchiectasis (white circle) and mild honeycombing (black arrows) on the background of ground glass opacity and coarse reticulation; (**E**) posteroanterior view and (**F**) lateral view chest radiographs show a perihilar and lower lung zone predominant reticular abnormality (black arrows); (**G**) posteroanterior view and (**H**) lateral view chest radiographs 18 years later demonstrate a reduction in lung volumes, and the prior interstitial opacities appear coarser and more prominent, particularly in the perihilar lung (white arrows).

Hard metal lung disease, also known as giant cell interstitial pneumonia, results from the inhalation of dust containing hard metals, most commonly tungsten carbide or cobalt, with workers usually being exposed during welding [54]. On CT, hard metal lung disease manifests as constrictive bronchiolitis with mosaic lung attenuation and air trapping visible on expiratory imaging [55]. Patients may also have superimposed ground glass opacities, small nodules, and occasionally consolidation [56]. Features of fibrosis can develop with chronicity, presenting as areas of reticulation, architectural distortion, and traction bronchiectasis (Figure 5) [55].

**Figure 6:**
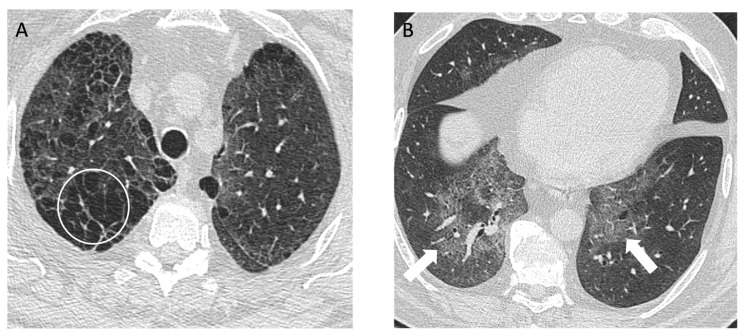

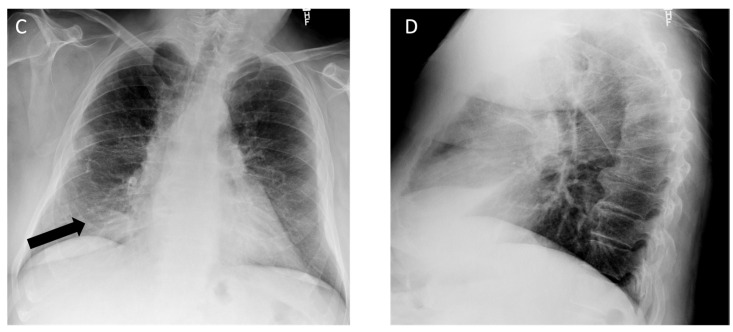
A 56-year-old male with a history of grinding aluminum for 5 years diagnosed with desquamative interstitial pneumonia (DIP) on surgical lung biopsy. There was a 10 pack year smoking history, but it was remote to the presentation, and the DIP was attributed to occupational exposure. (**A**) Axial computed tomography of the upper lung zone reveals emphysema (white circle), and the (**B**) basal lung zone demonstrates extensive ground glass opacity (arrows) with subtle small cysts or lucencies on close inspection; (**C**) posteroanterior and (**D**) lateral view chest radiographs 6 months later show persistent basilar opacities (black arrow).

Exposure to metals such as tungsten and rarely aluminum have also been reported to produce isolated desquamative interstitial pneumonia (Figure 6) [57]. On CT, desquamative interstitial pneumonia manifests as a lower lung zone predominance of consolidation and ground glass opacities with the formation of cystic air spaces. This inflammation can lead to fibrosis with traction bronchiectasis [58]. Cystic air spaces and adjacent traction bronchiectasis may persist even after the inflammation resolves.

### 4.5. Other Pneumoconioses

**Figure 7:**
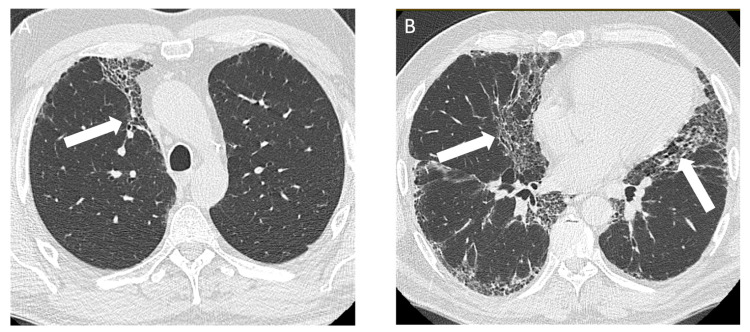
A 56-year-old male with a remote 20 pack year smoking history and a history of beryllium exposure who was working with electronics, diagnosed with chronic beryllium disease. Axial computed tomography shows interstitial fibrosis characterized by (**A**) peripheral subpleural and (**B**) peribronchovascular distribution of reticulation and mild traction bronchiectasis (arrows) with a basal predominance.

Chronic beryllium disease results from exposure to beryllium, a metal used in many modern industries, including metal welding, mining, and electronics [59]. Recently, there was a reported case of chronic beryllium disease in a Canadian metal welder, illustrating its persisting significance in modern times [60]. Chronic beryllium disease is a granulomatous condition that mimics sarcoidosis on CT, and many patients with chronic beryllium disease are indeed misdiagnosed with sarcoidosis in the absence of detailed occupational exposure history and blood beryllium lymphocyte proliferation testing (Figure 7) [60,61].

Talcosis results from the inhalation of talc powder, which is used in the manufacturing of cables, cosmetics, and many other products [62]. On CT, talcosis presents with small centrilobular nodules, ground glass opacities, and air trapping [63].

Siderosis results from the inhalation of iron dust. While imaging abnormalities may be present, siderosis is indolent and typically does not manifest in fibrosis [64]. However, patients who are exposed to iron dust may be exposed to other dusts such as silica and present clinically as silico-siderosis [65].

## 5. Hypersensitivity Pneumonitis

Hypersensitivity pneumonitis (HP) is an immune-mediated reaction to inhaled organic antigens and low-molecular weight inorganic particles [66]. The causative antigen cannot be identified in most cases, even with detailed occupational history; however, numerous cases are attributed to workplace exposure [67]. HP has been associated with countless antigens, and to this day, new associated antigens continue to be discovered [8]. For example, recently, there were reported cases of occupational HP from polyurethane adhesives in creating plant plug inserts [68], *Penicillium* spp. from preparing sausages and cheese [69], and polybutylene terephthalate from producing polyethylene terephthalate bottles [70]. There are some well-established workplace exposures leading to subtypes of HP, such as exposure to birds causing bird fancier’s lung and moldy hay or straw on farms causing farmer’s lung [8].

Metal cooling liquid (metal working fluid) is a particularly important exposure to be aware of in the industrial economy. Studies have shown a high prevalence of mycobacterial species and other micro-organisms present in metal cooling liquid mists; it is theorized that these antigen exposures cause HP, particularly in the absence of appropriate PPE [71].

**Figure 8:**
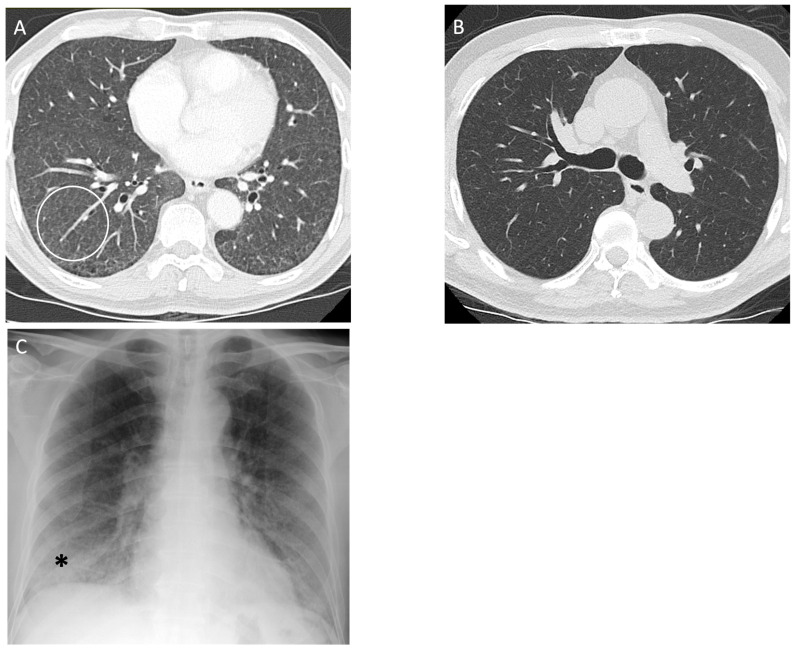
A 62-year-old non-smoking male with metal vapor exposure in the workplace was diagnosed with non-fibrotic hypersensitivity pneumonitis on surgical lung biopsy. (**A**) CT of lung bases demonstrates diffuse ground glass abnormality posteriorly (white circle); (**B**) follow up CT 6 months after being removed from the workplace demonstrates compete resolution of the opacities; (**C**) chest radiograph shows a subtle increase in lung density at the bases (asterisks) with relatively preserved lung volumes.

**Figure 9:**
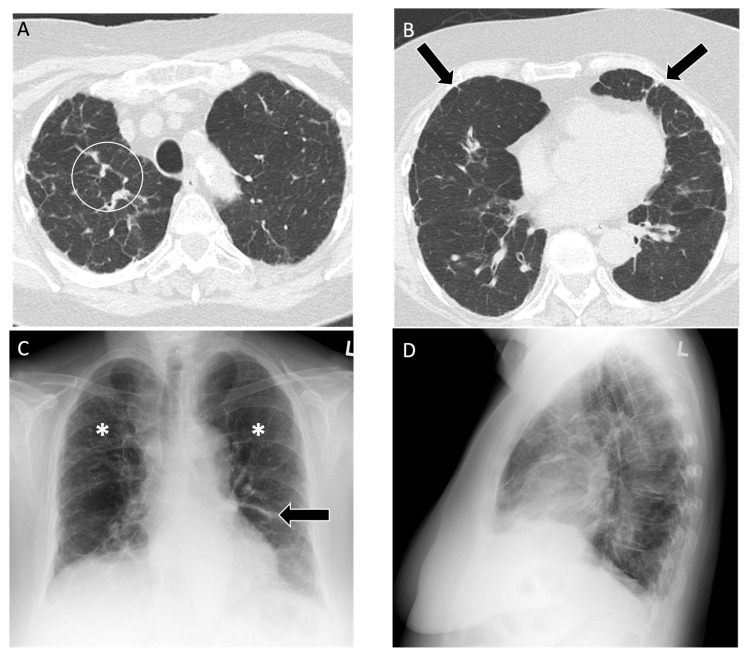
A 76-year-old non-smoking female was diagnosed with farmer’s lung, proven with biopsy. Computed tomography of the upper lobe demonstrates (**A**) peribronchovascular reticulations (white circle) with mosaic lung attenuation and (**B**) mild diffuse subpleural reticulations (black arrows); (**C**) posteroanterior and (**D**) lateral view radiographs demonstrate diffusely coarse reticulations and fibrosis (asterisks) in the upper lung zone with some mild volume loss. A staple line in left lower lobe is noted from a previous lung biopsy for diagnosis of farmer’s lung disease (black arrow).

CT findings of HP related to occupational lung disease are like those of HP from other exposures. HP may present in a non-fibrotic or a fibrotic fashion [66]. In non-fibrotic HP, there is a diffuse ground glass abnormality, potentially with nodules or consolidation, in combination with imaging evidence of small airways disease, such as centrilobular nodules, mosaic lung attenuation, and, on expiratory imaging, air trapping (Figure 8) [72]. Fibrotic HP classically presents with an upper lung zone and peribronchovascular predominant distribution of fibrosis (reticulation, traction bronchiectasis with or without honeycombing) in addition to CT evidence of small airways disease (centrilobular ground glass nodules, three-density pattern, or air trapping) (Figure 9) [67,73].

## 6. Imaging Evaluation

Many patients with suspected occupational lung disease will be initially evaluated with chest radiography. Although radiographs are often helpful, CT is needed for patients with abnormal chest radiographs or a high degree of clinical suspicion, such as those with abnormal pulmonary function tests despite a normal radiograph [5] (Table 1). A summary of imaging characteristics of different occupational lung diseases can be found in Table 2.

At our centre, all patients suspected of having occupational lung disease undergo the interstitial lung disease baseline protocol, as recommended by the Canadian Society of Thoracic Radiologists [74]. Generally, high-resolution CT (HRCT) of the chest is performed at end inspiration in thin sections (slice thickness ≤ 1 mm) with a sharp reconstruction kernel [75]. Prone views are helpful in evaluating the dependent lung, and we perform this in all patients with dependent abnormalities at baseline. Multiplanar reformats are useful in determining the craniocaudal distribution of disease, and maximum intensity projections are helpful to identifying diffuse nodules. We perform expiratory imaging in all patients at the baseline to evaluate air trapping. Prone and expiratory imaging is not performed routinely at follow-up. Intravenous contrast is not recommended in the routine evaluation of occupational lung disease.

**Table 1:** Clinical classification and approach to the diagnosis of occupational lung disease.

Occupational Lung Disease	Common Exposure Agents	Timing of Onset	Clinical Presentation	Clinical Diagnostic Testing	Imaging Evaluation
Work-Related Asthma	Mold, wood dust, flour, cement dust [11,12,13]	Sensitizer induced: latent onset; irritant induced: <24 h [15]	Wheezing, dyspnea, sneezing, nasal congestion [15]	Pulmonary function test or methacholine challenge [10]	Not needed for diagnosis, may be used to rule out other occupational diseases [16]
Pneumoconiosis	Silica, cobalt, asbestos, coal dust, talc dust, beryllium, iron dust [17,31,35,54,59,63,64]	Typically delayed 10–20 years [3]	Cough, dyspnea, pleuritic chest pain, fever, weight loss [18,53]	Potential exposure in history	Chest radiograph or HRCT non-contrast [75]
Hypersensitivity Pneumonitis	Bacteria, fungi, animal proteins, isocyanates [67]	Acute HP: 4–12 h after exposure; chronic HP: weeks-years [8]	Cough, dyspnea, fever [66]	Potential exposure in history, elevated IgG to antigen, inspiratory crackles, Bronchoalveolar lavage lymphocytosis [66]	Chest radiography or HRCT non-contrast [75]

**Table 2:** Characteristics of occupational lung diseases.

Occupational Lung Disease	Findings on Computed Tomography	Differentiating Features
Silicosis	Simple: small nodules in upper lung zone, pseudo-plaques, calcified lung nodules and/or lymph nodes [23,24] Complicated: conglomerate mass-like lesion, calcified lung nodules and/or lymph nodes, small peripheral lung nodules [25] Accelerated: similar to complicated silicosis [26]	Complicated: coalescence of silicotic nodules into a large mass [25] Accelerated: rapid progression (<10 years of exposure) [26]
Coal Worker’s Pneumoconiosis	Similar to simple or complicated silicosis, lymph node calcification is less common [34]	Difficult to differentiate by imaging only
Asbestos-Related Lung Disease	Pleural effusion, smooth pleural plaques, rounded atelectasis [42,43,44]	Presence of smooth pleural plaques, often bilateral and along all pleural surfaces [45,46]
Mesothelioma	Various types of pleural thickening, interlobar fissure involvement, pleural effusion, pleural plaques [51]	Atypical pleural plaques, chest wall invasion [51]
Hard Metal Lung Disease	Constrictive bronchiolitis, mosaic lung attenuation, air trapping, fibrosis with traction bronchiectasis [55,56]	Difficult to differentiate by imaging only; evidence of intersitial lung disease along with clinical history and histological examination supports diagnosis [55,56]
Hypersensitivity Pneumonitis	Non-fibrotic: diffuse ground glass opacities, nodules/consolidation present, centrilobular nodules [72] Fibrotic: peribronchiovascular fibrosis, small airway disease [67,72]	Difficult to differentiate by imaging only; evidence of ground glass opacities and air trapping along with bronchioalveolar lavage lymphocytosis aids diagnosis [66]

## 7. Conclusions

This pictorial review outlines the clinical and radiological approach to diagnosing occupational lung diseases. Key radiographic features to delineate disease subtypes are discussed. Old and new imaging examples of occupational lung diseases are provided to facilitate recognition of radiological features, which is crucial for early diagnosis and interventions to prevent further damage.

## Data Availability

No new data were created or analyzed in this study. Data sharing is not applicable to this article.
